# Noninvasive monitoring of central venous oxygen saturation by jugular transcutaneous near-infrared spectroscopy in pediatric patients undergoing congenital cardiac surgery

**DOI:** 10.3906/sag-1911-135

**Published:** 2020-08-26

**Authors:** Dilek ALTUN, Abdullah DOĞAN, Ahmet ARNAZ, Adnan YÜKSEK, Yusuf Kenen YALÇINBAŞ, Rıza TÜRKÖZ, Tayyar SARIOĞLU

**Affiliations:** 1 Department of Anesthesiology and Reanimation, Vocational School of Health Sciences,Acıbadem Mehmet Ali Aydınlar University, İstanbul Turkey; 2 Department of Cardiovascular Surgery, Acıbadem Bakırköy Hospital, İstanbul Turkey; 3 Department of Cardiovascular Surgery, Faculty of Medicine, Acıbadem Mehmet Ali Aydınlar University, İstanbul Turkey; 4 Department of Anesthesiology and Reanimation, Acıbadem Bakırköy Hospital, İstanbul Turkey; 5 Department of Pediatric Cardiovascular Surgery, Faculty of Medicine, Acıbadem Mehmet Ali Aydınlar University, İstanbul Turkey

**Keywords:** Near-infrared spectroscopy, central venous oxygen saturation, mixed venous oxygen saturation, congenital cardiac surgery

## Abstract

**Background and aim:**

In patients undergoing congenital cardiac surgery, it is crucial to maintain oxygen demand-consumption balance. Central venous oxygen saturation (ScvO_2_) is a useful indicator of oxygen demand and consumption balance which is an invasive method. Near-infrared spectroscopy (NIRS) is a noninvasive, continuous monitoring technique that measures regional tissue oxygenation. NIRS that is placed over the internal jugular vein cutaneous area (NIRSijv) has the potential to show ScvO_2_ indirectly. In this study, we aimed to determine the correlation between ScvO_2_ with NIRSijv in pediatric patients undergoing congenital cardiac surgery.

**Materials and methods:**

Fifty children participated in the study. Four patients were excluded for the inability of internal jugular vein (IJV) catheterization due to technical difficulties. After anesthesia induction, NIRS probes were placed on the IJV site with ultrasound guidance for the measurement of continuous transcutaneous oxygen saturation. The catheter insertion was also done through the IJV from the other side using ultrasound guidance. Cerebral oxygenation monitoring was done using NIRS with a single pediatric probe placed on the right forehead. Values of NIRSijv, cerebral NIRS (NIRSc) and ScvO_2_, were recorded at certain times until postoperative 24th hour.

**Results:**

Data were collected at 8 different time points. There was a significant correlation between ScvO_2_ and NIRSijv in all measurement time points (r = 0.91), (P = 0.001). The mean bias between ScvO_2_ and NIRSijv was 2.92% and the limits of agreement were from 11% to –5.2%. There was a moderate correlation between ScvO_2_ and NIRSc (r = 0.45), (P = 0.001). The mean bias between ScvO_2_ and NIRSc was 2.7% and the limits of agreement were from +26% to –20%.

**Conclusion:**

In this study, we found a strong correlation between ScvO_2_ and NIRS measurements taken from the internal jugular vein site. Accordingly, continuous noninvasive monitoring with transcutaneous NIRSijv can be an alternative method as a trend monitor for the central venous oxygen saturation in pediatric cardiac patients undergoing congenital cardiac surgery.

## 1. Introduction 

Monitoring of mixed venous oxygen saturation (SvO_2_) in pediatric cardiac surgery patients is one of the most critical issues which reflects the balance between tissue oxygen supply and demand and also a good indicator of cardiac output (CO) [1–2]. However, oxygen delivery (DO_2_) cannot alone reflect the tissue perfusion, oxygen consumption (VO2) should also be known. Because of this fact, venous oxygen saturation becomes more important for monitoring of the CO [1–2].

On the other hand, monitoring of SvO_2_ is possible generally with invasive measurement techniques like pulmonary artery catheterization. Instead of SvO_2_ measurement via pulmonary artery catheterization because of technical difficulties and possible complications, central venous oxygen saturation (ScvO_2_) measurements obtained from internal jugular catheters can be used to identify ScvO_2_ besides its benefits with lower cost, time savings and lower risks than pulmonary artery catheterization [1–5].

Since ScvO_2_ monitoring is invasive, which restricts its clinical use, the use of noninvasive methods has been developed which is very important for patients undergoing congenital cardiac surgery, especially for low weight patients because children undergoing cardiac surgery are at increased risk for decreased perfusion and oxygen delivery in the intraoperative and postoperative periods [6–8].

Near-infrared spectroscopy (NIRS) is a noninvasive continuous monitoring technique for detecting regional tissue oxygen saturation (rSO2). It detects hemodynamic changes earlier than any single venous saturation measurement [4–8].

Changes in regional oxygen saturation obtained with jugular transcutaneous NIRS have been found to have a close correlation with changes in ScvO_2_ by several studies which were generally held in adult patients [8–10]. However, there is limited data regarding the correlation between NIRS that is placed over the internal jugular vein cutaneous area (NIRSijv) and ScvO_2_ in pediatric cardiac surgery patients [6–10].

This study aimed to evaluate the relationship between central venous oxygen saturation obtained through the central venous catheter inserted into the internal jugular vein (IJV) with tissue oxygen values obtained with NIRSijv in pediatric patients undergoing congenital cardiac surgery. 

## 2. Materials and methods 

After obtaining University Ethics Committee (2018-4/11) approval and taken written informed consent of parents, 50 pediatric patients who received general anesthesia for elective congenital cardiac operations with cardiopulmonary bypass were enrolled in this prospective, observational study. Patients with noncyanotic congenital heart pathologies without intracardiac mixing were included in the study to obtain a relatively homogenous cohort of patients. Exclusion criteria were; absence of written informed consent, emergency operations, contraindication or failure to the cannulation of the right internal jugular vein, ventricular assist device or extracorporeal membrane oxygenation support, patients in whom data variables could not be simultaneously recorded because of technical problems. Standard monitoring was done for every patient. After induction of general anesthesia, tracheal intubation, and radial or femoral artery cannulation, a central venous catheter (CVC) was inserted into the superior vena cava (SVC) through the right internal jugular vein with the guidance of ultrasound (US) (FUJIFILM SonoSite Inc., Bothwell, WA, USA). Following induction of anesthesia, NIRS sensors (Covidien Products, Medtronic Minimally Invasive Therapies, Minneapolis, MN, USA) were placed on the internal jugular vein site after finding the proper place of the left internal jugular vein with the guidance of US to all patients for the measurement of continuous transcutaneous oxygen saturation. Cerebral oximetry (rSO2) was measured using NIRS with a single pediatric probe placed on the forehead approximately 0.5 cm above the eyebrow with the medial edge at the midline of the forehead (NIRSc).

The technique of anesthesia was standardized for all patients. Cardiopulmonary bypass (CPB) was instituted in a standard manner by cannulating inferior vena cava, superior vena cava and ascending aorta. After the operation, patients were transferred to the cardiac intensive care unit (CICU).

Demographic characteristics, diagnosis, surgical procedure, cardiac bypass time and cross-clamp time were recorded. To obtain ScvO_2_, blood was withdrawn from the distal port of the double lumen CVP catheter. Blood gas samples in heparinized 2 mL syringes were analyzed within 60 s from withdrawal, using a blood gas analyzer (ABL 800 Flex; Radiometer).

Data were collected after anesthesia induction (T1), 10th min of CPB (T2), after CPB (T3), postoperative 2nd h (T4), postoperative 6th h (T5), postoperative 12th h (T6), postoperative 16th h (T7), postoperative 24th h in CICU (T8). At each time points ScvO_2_, SaO_2_, SpO_2_, NIRSijv, and NIRCc values were recorded.

### 2.1. Statistical analysis 

Statistical analysis was performed using IBM SPSS Statistics 22.0 (IBM Corp., Armonk, NY, USA). Descriptive statistics were expressed as mean, standard deviation, and frequency. Shapiro-Wilks test was used to evaluate the conformity of the parameters to a normal distribution. Pearson’s correlation analysis was used to analyze any correlation among variables. The Bland-Altman analysis for repeated measures was used to further assess the similarity of methods, and a range of agreement was defined as mean bias and 95% confidence interval (CI). To evaluate reproducibility, the intraclass correlation coefficient (ICC) (2-way mixed, absolute agreement) of the various parameters between both measurement sessions was assessed. The ICC can be arbitrarily interpreted as poor (< 0.20), fair (0.21–0.40), moderate (0.41–0.60), good (0.61–0.80) and excellent (0.81–1.00). A P-value of < 0.05 was considered statistically significant.

At the beginning of the study, a pilot study was conducted with 10 patients. Accordingly, the minimum required number was found to be 34 at 0.05 and β = 0.20 levels.

## 3. Results 

### 3.1. Patient characteristics

Fifty pediatric patients were included in the study. Four patients were excluded for the inability of internal jugular vein catheterization due to technical difficulties. Data from the remaining 46 pediatric patients were included in the statistical analysis. Demographic data and congenital cardiac defect type of the patients are presented in Table 1.

**Table 1:** Demographic data and congenital cardiac defect type of the patients.

Age (months)	67.6 ± 44.2 (7–174)	
Weight (kg)	14 ± 7.3 (4.2–31.5)	
Sex (Female/Male)	31/15 (67%/33%)	
Congenital cardiacdefect type	VSD	20 (43%)
	ASD	9 (19.5%)
	CAVSD	6 (13)
	AI	6 (13)
	PS	3 (6.5)
	MS	2 (4.3)

Data were given as mean ± standart deviation (SD) or number of patients (% incidence). VSD: Ventricular septal defect; ASD: Atrial septal defect; CAVSD: Complete atrioventricular septal defect; AI: Aortic interruption; PS: Pulmonary stenosis; MS: Mitral stenosis.

### 3.2. Clinical outcomes

There was a statistically significant correlation at an average of 0.91 between ScvO_2_ and NIRSijv in all measurement time points (P = 0.001) (Table 2).

**Table 2:** Correlations of all measurements.

		NIRSijv (r/p)	NIRSc (r/p)
T1 After induction	ScvO_2_	0.925/0.001	0.479/0.001
	SaO_2_	0.456/0.001	0.230/0.128
	SpO_2_	0.475/0.001	0.198/0.193
T2 CPB 10th min	ScvO_2_	0,960/0.001	0,167/0,266
	SaO_2_	0.230/0.123	0.138/0.361
	SpO_2_	–0.061/0.686	–0.274/0.066
T3 after CPB	ScvO_2_	0.901/0.001	0.468/0.01
	SaO_2_	0.435/0.003	0.382/0.009
	SpO_2_	0.384/0.008	0.394/0.007
T4 postop 2nd h	ScvO_2_	0.881/0.001	0.357/0.015
	SaO_2_	0.284/0.056	0.396/0.006
	SpO_2_	0.267/0.073	0.353/0.016
T5 postop 6th h	ScvO_2_	0.916/0.001	0.609/0.001
	SaO_2_	0.474/0.001	0.504/0.001
	SpO_2_	0.336/0.023	0.396/0.006
T6 postop 12th h	ScvO_2_	0.934/0.001	0.645/0.001
	SaO_2_	0.528/0.001	0.414/0.004
	SpO_2_	0.441/0.002	0.414/0.004
T7 postop 18th h	ScvO_2_	0.892/0.001	0.574 / 0.001
	SaO_2_	0.379/0.009	0.349/0.017
	SpO_2_	0.389/0.007	0.372/0.011
T8 postop 24th h	ScvO_2_	0.931/0.001	0.496/0.001
	SaO_2_	0.416/0.004	0.523/0.001
	SpO_2_	0.287/0.053	0.418/0.004

Pearson correlation analysis. P value < 0.05 is considered as a statistically significant difference.NIRSijv: Near infrared spectroscopy internal jugular vein; NIRSc: Near infrared spectroscopy cerebral; CPB: Cardiopulmonary bypass.

While there was a statistically significant correlation between ScvO_2_ and NIRSijv in all measurement time points; arterial oxygen saturation (SaO_2_) showed a moderate correlation with NIRSijv at T1, T3, T5, T6, T7, T8 times (0.33) (P = 0.001); and there was no correlation at T2, T4 times (P > 0.05). Peripheral oxygen saturation (SpO_2_) also showed a moderate correlation with NIRSijv at T1, T3, T5, T6, T7 times (0.25) (P = 0.001); and there was no correlation at T2, T4, T8 times (P > 0.05) (Table 2).

Cerebral NIRS showed a moderate correlation with ScvO_2_ at all measurement time points except T2 time (r = 0.45) (P = 0.001). Peripheral oxygen saturation (SpO_2_) and SaO_2_ showed a moderate correlation with NIRSc at T3, T4, T5, T6, T7, T8 (0.32, 0.29) (P = 0.001); there was no correlation at T1 and T2 times (P > 0.05) (Table 2, Table 3). 

**Table 3:** NIRSc, NIRSijv, ScvO_2_ and Hb values in all time points.

	NIRSc	NIRSijv	ScvO_2_	Hb
	mean ± SD (min–max)	mean ± SD (min–max)	mean ± SD (min–max)	mean ± SD (min–max)
T1 after induction	65.8 ± 10 (41–86)	67.15 ± 8.79 (45–83)	65.2 ± 10.16 (45–83)	12.5 ± 1.9 (10.8–13.5)
T2 CPB 10th min	61.3 ± 11.5 (39–87)	69.74 ± 11.85 (40–87)	67.96 ± 14.93 (40–87)	8.8 ± 0.1 (8.7–8.9)
T3 after CPB	61.5 ± 12.8 (37–89)	63.91 ± 10.83 (45–89)	60.87 ± 12.14 (45–89)	10.6 ±1.6 (9.5–11.8)
T4 postop 2nd h	68.3 ± 11.4 (32–89)	64.91 ± 8.02 (44–82)	61.17 ± 8.89 (44–82)	12.1 ± 0.3 (11.9–12.3)
T5 postop 6th h	66 ± 10.4 (43–88)	63.76 ± 8.11 (42–80)	60.48 ± 8.78 (42–80)	11.7 ± 1.3 (10.8–12.7)
T6 postop 12th h	66.4 ± 10.3 (47–88)	65.43 ± 8.12 (41–80)	61.63±8.44 (41-80)	11.8 ± 0.3 (11.6–12.1)
T7 postop 18th h	68.5 ± 11.1 (49–91)	66.48±9.26 (42-81)	63.76 ± 9.06 (42–81)	11.1 ± 0.3 (10.9–11.4)
T8 postop 24th h	69.3 ± 8.6 (53–85)	66.91 ± 7.72 (48–83)	64.09 ± 8.92 (48–83)	11.5 ± 0.3 (10.6–11.8)

NIRSc: Near infrared spectroscopy cerebral; NIRSijv: Near infrared spectroscopy internal jugular vein; ScvO_2_: Central venous oxygen saturation; Hb: Hemoglobin; CPB: Cardiopulmonary bypass.

The mean bias between ScvO_2_ and NIRSijv was 2.92% and the limits of agreement were from 11% to –5.2% (Figure a). The mean bias between ScvO_2_ and NIRSc was 2.7% and the limits of agreement were from +26% to –20% (Figure b). 

**Figure a:**
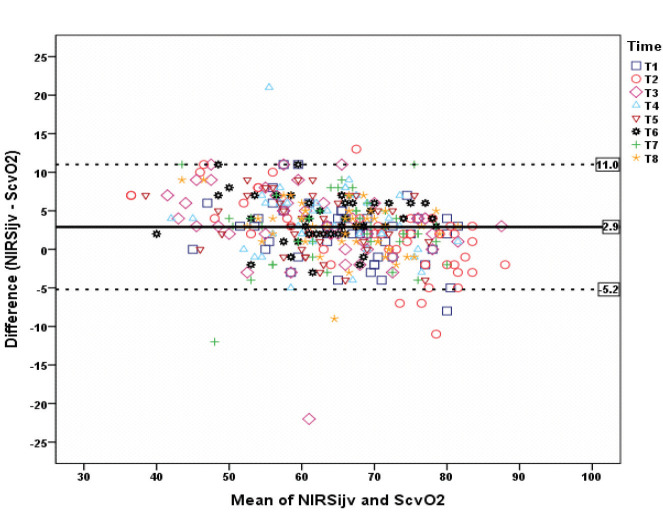
Bland Altman analysis of NIRSijv and ScvO_2_ at all times.

**Figure b:**
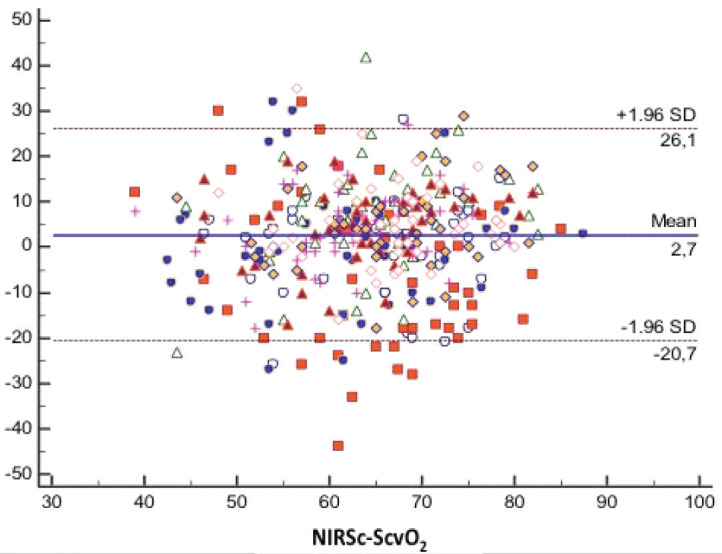
Bland Altman analysis of NIRSc and ScvO_2_ at all times.

The difference between NIRSijv and ScvO_2_ values was statistically significant in all measurement time intervals (P < 0.05). There was a positive strong correlation between the two measurements at all times (P < 0.05). Correlation levels ranged from 0.881 to 0.960.

When the intraclass correlation levels were examined, it was found that there was no agreement between the measurements at T2 time. At other times, numbness was observed, but poor for T4, fair for T1, T3, T5, T6, T7, T8 (Table 4).

**Table 4:** Correlation (r) between NIRSijv and ScvO_2_.

	NIRSijv	ScvO_2_	Correlation		Agreement
	mean ± SD	mean ± SD	^a^r	p	^b^ICC (95% CI)
T1 after induction	67.15 ± 8.79	65.20 ± 10.16	0.925	<0.001	0.477 (0.218, 0.673)
T2 CPB 10th min	69.74 ± 11.85	67.96 ± 14.93	0.960	<0.001	0.150 (-0.117, 0.407)
T3 after CPB	63.91 ± 10.83	60.87 ± 12.14	0.901	<0.001	0.471 (0.210, 0.669)
T4 postop 2nd hour	64.91 ± 8.02	61.17 ± 8.89	0.881	<0.001	0.281 (0.004, 0.522)
T5 postop 6th hour	63.76 ± 8.11	60.48 ± 8.78	0.916	<0.001	0.551 (0.277, 0.734)
T6 postop 12th h	65.43 ± 8.12	61.63 ± 8.44	0.934	<0.001	0.567 (0.262, 0.755)
T7 postop 18th h	66.48 ± 9.26	63.76 ± 9.06	0.892	<0.001	0.509 (0.227, 0.705)
T8 postop 24th h	66.91 ± 7.72	64.09 ± 8.92	0.931	<0.001	0.426 (0.120, 0.649)

^a^Pearson correlation coefficient, ^b^ICC (Intraclass correlation coefficient). T1-T3 indicate perioperative phase, T4-T5 indicate early postoperative phase and T6-T8 indicate late postoperative phase. NIRSijv: Near infrared spectroscopy internal jugular vein; ScvO_2_: Central venous oxygen saturation; CPB: Cardiopulmonary bypass.

## 4. Discussion 

In this study, we investigated the relationship between ScvO_2_ measured from blood gas samples taken directly from IJV and regional transcutaneous oxygen saturation measured by noninvasive measurement technique NIRS at the internal jugular venous cutaneous area. We have found a strong positive correlation between ScvO_2_ and NIRSijv measurements at every time point. While there was a strong correlation between ScvO_2_ and NIRSijv measurements; there was only a moderate correlation between the ScvO_2_ and NIRSc measurements.

The demonstration of a strong correlation between NIRSijv and ScvO_2_ would allow the clinician to use noninvasive monitoring techniques for ScvO_2_ which is very important to estimate the balance between tissue oxygen delivery, consumption and so CO. Continuous monitoring of ScvO_2_ would represent a major advance for clinicians to detect physiological changes in the oxygen consumption of underlying tissue which will enable us to estimate life-threatening events earlier. Also, using a noninvasive continuous technique for monitoring ScvO_2_, will decrease iatrogenic complications such as infection, embolization, and iatrogenic anemia with a decreased number of blood samples taken from the pediatric patients which are very crucial especially for low weight pediatric patients [6–11].

NIRS is used for routine monitoring of cerebral oxygenation, early prediction of neurological disorders for pediatric patients undergoing congenital cardiac surgery [12–14]. In the literature, various studies demonstrated the correlation between NIRSc with ScvO_2_. Ranucci et al. demonstrated the relationship between NIRSc and ScvO_2_ in pediatric patients undergoing cardiac operations. According to their study, during cardiopulmonary bypass many times, but not always, there was a positive relationship between NIRSc and ScvO_2_. However, in their study, there was a constant positive bias (ScvO_2_ values were higher than NIRSc values) of 5.6%, with a precision of 10.4% [15].

Tortoriello et al. studied the correlation between ScvO_2_ that they have taken from the pulmonary artery catheter or superior vena cava catheter placed intraoperatively, with NIRSc in pediatric cardiac surgery patients. In their study, they have found a strong correlation between NIRSc and ScvO_2_ (r: 0.67; P < 0.0001), because of the wide limits of agreement they concluded that it may not be possible to predict absolute values of ScvO_2_ on the noninvasive measurement of NIRSc [1]. On the other hand, similar to our study findings, Marimon et al. and Ricci et al. found a moderate correlation between NIRSc and ScvO_2_ in pediatric patients undergoing congenital cardiac surgery (r = 0.58, P < 0.008; r = 0,37; P < 0.001, respectively) [16–17]. Dullenkopf et al. found a moderate correlation of r = 0.33 between the SvO_2_ and postoperative NIRSc values of adult patients experienced cardiac surgery. Concerning their findings, they hypothesized that this might not correctly represent exact saturation, but might be acceptable [18]. This study differs from our study with its patient population. Our study included only pediatric patients undergoing cardiac surgery. 

These studies, with wide limits of agreement, suggest that it may not be possible to predict the absolute values of ScvO_2_ and since they suggested that NIRSc is weakly correlated with direct measurements of ScvO_2_, based on these results, instead of NIRSc we investigated the correlation between NIRSijv and ScvO_2_ among pediatric patients undergoing congenital cardiac surgery. There are only limited studies investigating the correlation between NIRSijv and ScvO_2_. However, our study showed a significant correlation between NIRSijv and ScvO_2_ (P < 0.05) and the limits of agreement were from 11% to –5.2%. To our knowledge, this is the first study that shows a high correlation between NIRSijv and ScvO_2_ among pediatric patients undergoing congenital cardiac surgery. Similarly, Colquhoun et al. investigated the correlation of two noninvasive technologies (reflectance plethysmography and NIRS) for the estimation of regional venous saturation by using venous blood gas analysis as a gold standard. According to their study, the mean biases between venous blood gas analysis and NIRS were 10.8% and 2.0% for NIRSc and NIRSijv, respectively. The limits of agreement were from 33.1% to –11.4% and from 19.5% to –15.5% for NIRSc and NIRSijv, respectively. Both sites showed a weak but statistically significant correlation with the measured saturation (r2 = 0.22, 0.28 and P = 0.04, 0.03 for NIRSc and NIRSijv, respectively) [9]. This study differs from our study with wide limits of agreements, and the study included adult patients undergoing cardiac surgery. Ruan et al. also investigated the correlation of NIRSijv with ScvO_2_ and described a range of the mean ± 1.96 SD of the difference between the NIRSijv and ScvO_2_ were 3 ± 10.2, and the confidence interval of the difference between the NIRSijv and ScvO_2_ was from –7.2% to 13.2% for 13 adult patients (aged > 18) who required surgery because of illness (except for neck surgery) [10]. This study with its results and methodology is similar to our study, but the difference from our study is that it includes adult patients who have surgery rather than cardiac surgery. In our study, we found a statistically high correlation between NIRSijv and ScvO_2_ (r: 0.91) while there was only a moderate correlation between NIRSc and ScvO_2_ (r: 0.47). 

In some instances, NIRSijv may be an advantageous method instead of NIRSc. For example, intracranial pathologies can compromise the values of NIRSc measurement [19, 20]. With the effect of hypoxia or intracranial pathologies (hemorrhage, ischemia, etc.) the difference between jugular venous oxygen saturation and cerebral oxygenation may increase. If the NIRS probe is placed on the affected area of the brain, low values can be obtained. Moreover, high values can be obtained if the NIRS probes are placed in the nonaffected area. Because of the cerebral autoregulation, blood flow and oxygen delivery to the brain can be maintained in low cardiac output situations. Measurements from the cerebral cortex especially at a specific site on the frontal cortex are usually weakly correlated compared to the direct measurements of jugular venous oxygen saturation. This can be also related to the fact that regional oxygen saturation measurements taken only at one point in the brain which may or may not represent global oxygen supply-demand matching. In these areas, the distribution of arterial and venous blood can change due to the changes in systemic venous resistance, mechanical ventilatory support parameters, although arterial saturation and regional oxygen venous saturation remain stable [9, 10, 19, 20]. 

Various studies have validated the accuracy of NIRSc comparison to IJV oxygen saturation [20–25]. On the other hand, the use of the NIRS algorithm which is validated for cerebral tissue oxygenation can be questioned for use in solely venous oxygenation as this algorithm is based on the assumption that cerebral tissue contains arterial and venous blood distribution in a 1:3 ratio [24]. However, cerebral tissue can show large variations in contribution to changing values of venous contribution from 60% to 100% [21, 25]. 

The documented presence of close correlation with NIRSc and jugular bulb oxygen saturation and central SvO_2_ in other pediatric studies support the fact that NIRS is predominantly affected by venous contribution [1, 13–15, 17]. Since the NIRS measurement depends on the absorption of infrared light by oxyhemoglobin and deoxyhemoglobin it would give the measurements of the underlying tissue of IJV where it reflects the only venous side. So, direct measurements with NIRSijv will give directly venous measurements as well.

The principle of NIRS monitoring depends on the absorbance of different near-infrared light wavelengths with changes in the concentration of an absorber that can pass through skin, soft tissue and bone with relative ease. Penetration into the cortex is possible up to 0.5 cm in adults, 1.5 cm in infants and several centimeters in preterm newborns at a standard 3 cm source-detector separation [20, 26–28]. Therefore, with contrast to the adults, we can take more accurate measurements in pediatric patients due to the thinner tissue thickness [21, 29–31]. Since our study population consists of only pediatric patients, it is very crucial for clinicians to have the opportunity of obtaining more accurate results when compared to the studies with adult patients. The second important point is the use of the US to find the proper place of IJV and reduce the interference from the carotid artery and near tissues to increase the reliability and value of the measurements in our study. 

There were some limitations to our study. Firstly, we recorded the measurements of NIRSijv on an hourly basis. Since NIRS is a continuous monitoring technique, recording the measurements only with an hourly basis may limit the usefulness of this monitoring. Secondly, inaccurate measurements resulting from artifacts can prevent accurate evaluation of NIRS outcomes. Another limitation of our study was the small patient number with a limited population, although the sample size was obtained by power analysis. Also, in our study, only patients with acyanotic congenital cardiac disease were included. However, a significant number of congenital heart surgery patients have cyanotic cardiac defects. It is uncertain how NIRS measurements would be in cyanotic patients. Studies with larger patient groups including both cyanotic and acyanotic patients are needed for further conclusions.

Transcutaneous NIRS cannot replace ScvO_2_ monitoring in cardiac surgery patients. However, NIRSijv measurements may provide valuable information about the regional circulation which can help in the early management of pediatric patients undergoing congenital cardiac surgery. In addition to other monitoring techniques such as SpO_2_, SaO_2_, continuous follow-up of transcutaneous venous oxygen saturation by NIRS technique is a reliable method and enables accurate and immediate measurements and follow-up of tissue oxygenation. Using a noninvasive continuous technique for monitoring ScvO_2_ will decrease iatrogenic complications such as infection, embolization, and iatrogenic anemia with a decreased number of blood samples taken from the pediatric patients which are very crucial especially for low weight pediatric patients. 

This study suggests that placement of NIRS probes directly over IJV appeared to approximate internal jugular venous saturation (lower mean bias and tighter limits of agreement) and may provide valuable information of regional circulation compared to NIRSc. A timely diagnosis of low oxygen delivery might improve critical clinical conditions by leading way to immediate therapy. These findings suggest that NIRSijv may offer information regarding oxygen supply and demand matching which gives clinicians accurate results for predicting the ScvO_2_.

## Acknowledgment/Disclaimers

Presented in part at 19th National Intensive Care Congress, 19-22th April 2018, Antalya, Turkey (Oral presentation/Second Prize in Clinical Study Oral Presentation Competition). Any specific grant from funding agencies in the public, commercial or not for profit sectors has not been received for this research.

## Informed consent

The study protocol received institutional review board approval (University Ethics Committee Number:2018-4/11) and that all participants provided informed consent in the format required by the relevant authorities and/or boards.
